# German Law Reform Does Not Reduce the Prevalence of Coercive Measures in Residential Institutions for Children, Adolescents, and Young Adults With Intellectual and Developmental Disabilities

**DOI:** 10.3389/fpsyt.2021.765830

**Published:** 2021-10-28

**Authors:** Julia M. Geissler, Elisabeth Werner, Wolfgang Dworschak, Marcel Romanos, Christoph Ratz

**Affiliations:** ^1^Department of Child and Adolescent Psychiatry, Psychosomatics and Psychotherapy, Center of Mental Health, University Hospital of Wuerzburg, Wuerzburg, Germany; ^2^Chair of Special Education IV–Education for People With Intellectual and Developmental Disabilities, University of Wuerzburg, Wuerzburg, Germany; ^3^Chair of Special Education–Education for People With Developmental and Intellectual Disabilities University of Regensburg, Regensburg, Germany; ^4^German Centre of Prevention Research in Mental Health, University Wuerzburg, Wuerzburg, Germany

**Keywords:** intellectual disabilities, developmental disabilities, challenging behaviours, employee stress, coercive measures, residential institutions

## Abstract

**Background:** Approximately 10% of children, adolescents and young adults with an intellectual and developmental disability (IDD) in Bavaria live in residential institutions. 2015 saw media reports raising suspicions about excessive use of coercive measures (cM) in those institutions. Until a law reform at the end of 2017 made permission from family courts mandatory for cM, their use was governed by parental consent. The REDUGIA project conducted a representative survey comparing cM and their relation to challenging behaviour (cB) and employee stress in Bavaria pre and post reform.

**Methods:** We sent questionnaires to 65 residential institutions for children, adolescents and young adults with IDD in 2017 (pre reform, T1) and 2019 (post reform, T2). To assess changes, we analysed data from all available questionnaire pairs (T1 and T2, *N* = 43). We calculated paired *t*-test and correlative analyses concerning the relationship between cB, cM, and employee stress.

**Results:** The number of residents overall (T1: *N* = 1,661; T2: *N* = 1,673) and per institution (T1: *m* = 38.6 ± 32.0; T2: *m* = 38.9 ± 34.5, *p* = 0.920) remained stable. We did not see any changes in the *Index cB* (*p* = 0.508) or the proportion of residents per institution displaying various types of challenging behaviour (all ps>0.220). There was no change in the *Index cM* (*p* = 0.089) or any indicator of employee stress, all ps > 0.323. At follow-up, the *Index cB* correlated positively with the *Index cM* (*r* = 0.519 *p* < 0.001). Regarding employee stress, the *Index cB* correlated positively with the frequency of sick leave (*r* = 0.322, *p* = 0.037) and physical attacks on employees (*r* = 0.552, *p* < 0.001). The *Index cM* also correlated positively with the frequency of sick leave (*r* = 0.340, *p* = 0.028) and physical attacks on employees (*r* = 0.492, *p* = 0.001).

**Discussion:** Coercive measures are not a general phenomenon, but are focused on specialised institutions. The law reform did not lead to changes in the number of children, adolescents and young adults with IDD affected by coercive measures in residential institutions in Bavaria. There were still large discrepancies between institutions in the prevalence of challenging behaviour and coercive measures. Coercive measures were associated with challenging behaviour and employee stress. Taken together, findings from REDUGIA emphasise the need to prevent challenging behaviour and thus coercive measures.

## Introduction

Approximately 1% of the population fulfil criteria for an intellectual and developmental disability (IDD; IQ <70) (1). Youths with IDD are at an increased risk for psychiatric disorders, with 40–50% compared to 10% in the general population in this age group having at least one psychiatric diagnosis (1, 2). At the same time, psychiatric comorbidity is underdiagnosed in this group, because symptoms are a) not reported due to limited communication skills (underreporting), b) primarily attributed to the IDD (diagnostic overshadowing) or c) retrospectively reported as having always been present (baseline exaggeration) (3, 4).

These undiagnosed and untreated psychiatric disorders contribute to the display of challenging behaviour. Around 52% of school-aged children with IDD show challenging behaviour (cB) (5), with self-injury and aggression towards others being the most problematic (6, 7). Challenging behaviour, especially aggressive behaviour, is one of the major issues both professional and family caregivers of people with IDD face, bringing with it a heightened risk to mental health and quality of life (8–12). We recently reported that challenging behaviour is linked to the use of coercive measures (cM) (13). Assuming a direct causal relation, the use of coercive measures may be avoided if the underlying causes of the challenging behaviour were addressed—either by treating relevant psychiatric disorders or by shaping the environment and providing the children with alternative strategies to get their needs met.

In 2011, the Winterbourne View inquiry exposed the inhumane treatment of people with learning disabilities showing challenging behaviour at hospitals run by a private company in the UK. This cast a spotlight on systemic problems leading to a lack of protection for this vulnerable population. A serious case review was commissioned, addressing the causes and laying out preventative measures. In 2015, German media alleged an excessive and inappropriate use of coercive measures in institutions for children, adolescents and young adults with IDD. In response, the Bavarian State Ministry of Labour and Social Welfare, Family Affairs and Women (StMAS) initiated an *ad hoc* examination, which found no indication of systematic abuse of coercive measures (14). An expert commission appointed by the ministry recommended a 10-point plan to improve and ensure quality standards in institutions for children and adolescents (15). The StMAS furthermore funded the SEKiB research consortium (16, 17) comprising three research projects on the reduction of coercive measures. Since there was a lack of data concerning the prevalence of challenging behaviour and coercive measures in institutions for children, adolescents and young adults with IDD, the REDUGIA project (Reducing Coercive Measures on Children and Adolescents with Intellectual and Developmental Disabilities) aimed to assess the magnitude of the issue by conducting a comprehensive survey.

In Germany, §1631b BGB governs the use of coercive measures. In the original version of the law, freedom-restricting measures (‘freiheitsentziehende Maßnahmen’) were defined as an all-encompassing restriction of a person's freedom of movement (e.g., in a psychiatric clinic or a restricted section in a residential institution). Those measures required permission from the family courts. However, freedom-limiting measures (‘freiheitsbeschränkende Maßnahmen’) based on §1631b were defined as any measure that is appropriate and common considering the child's age and the circumstances of their residential situation and is within the framework of general duties of education and supervision. Those measures did not require permission from the courts, since they were of shorter duration and limited to the respective situation. They did however require parental permission. Since the only distinction between freedom-restricting and freedom-limiting measures arose from subjective factors such as intensity, duration and child's age, this resulted in a sliding scale.

Since the amendment in October 2017, §1631b BGB defines coercive measures (‘freiheitsentziehende Maßnahmen’) broadly as any measure that restricts a child's freedom of movement against their will over long periods of time with medication, mechanical or any other means, which they cannot overcome without assistance. The frequency/duration criterion is met when the coercive measure is employed either for more than 24 h, occurs regularly at certain times or in certain situations, or is used repeatedly. For the use of coercive measures, institutions are now required to obtain permission and supervision from the family courts. Certain types of coercive measures (e.g., helmets, protective clothing) are not regulated by the law and still only require parental permission.

It is important to note that when a child's behaviour constitutes a danger to themselves or to others, the emergency use of coercive measure is permitted. Still, institutions must apply for retroactive permission from the court.

To our knowledge, there are no systematic evaluations across larger catchment areas in other countries on the prevalence of coercive measures across (1) residential homes, institutions or facilities for (2) children, adolescents and young adults with (3) intellectual disabilities.

In the following section, we provide a summary of the literature meeting at least two criteria. We only included studies conducted on larger areas, not just from one facility.

We identified three studies on the general use of coercive measures in people with disabilities by either service providers or authorities. Saloviita et al. (18) surveyed all adult residents with intellectual disabilities in a single special care district in Finland. They found high rates of challenging behaviour (72%), with 56% of instances of challenging behaviour being met with a negative intervention (18). Saloviita et al. (19) further conducted a postal survey on the total population of children in Finland aged 5–15 years entitled to the highest disability allowance with their first three diagnoses from the ICD-10 categories F7 (Intellectual disabilities), F8 (Pervasive and specific developmental disorders) or F9 (Behavioural and emotional disorders with onset usually occurring in childhood and adolescence). The authors received a response from 25.9% of families. Of those, 22% (*N* = 54) reported that their child had been restrained, secluded or subjected to aversive procedures by authorities. However, only families where the child was living at home were included (19). Webber et al. (20) attempted to collect population-based date on the use of chemical and mechanical restraint and seclusion ‘when a person was in receipt of a disability service’ in the State of Victoria, Australia. The mean age of the sample was 36 ± 15.6 years. The authors found that 9% of people with an IDD who received a government-funded disability support service were subject to at least one of those coercive measures. Psychiatric morbidity increased the risk of coercive measures (20).

Regarding the use of coercive measures in residential facilities for people with intellectual disabilities, we found studies from Sweden, Finland and the UK. Emerson et al. (21) investigated the use of physical restraint, mechanical restraint, sedation and seclusion in UK adults with intellectual disabilities receiving various types of residential supports by drawing small samples from village communities, NHS residential campuses and community-based dispersed housing schemes. They found between 3% (mechanical restraint) and 44% (physical restraint) to be affected by coercive measures (21). Lundström et al. (22) found that in a convenience sample of people with ID (16–90 years) living in 118 group homes in one county in northern Sweden, 17.8% of residents had experienced physical restraint, especially those with more behavioural issues or physical impairments. However, in Sweden the use of coercive measures is not permitted, so that number is still high (22). Saloviita et al. (19) cited a study conducted in Finland ‘by an official state monitoring agency, Valvira, [which] found only occasional examples of the use of coercive measures in their study of 69 residential organisations for people with intellectual disabilities (23).’ However, since the Valvira study is only available in Finnish, we were unable to assess the quality or report specific numbers.

There are several international studies on the use of coercive measures in psychiatric settings for children and adolescents. In their literature review on the prevalence of seclusion and restraint in children and adolescents (<21 years) treated in a psychiatric setting in the last 10 years, De Hert et al. (24) identified 7 studies conducted in the US, Australia and Finland. They reported an overall baseline rate of 26–29% of patients to experience those types of coercive measures. In Finland, Ulla et al. (25) conducted a register study on the use of exclusion and restraint in all adolescents aged 12–17 years who received psychiatric inpatient treatment between 1996 and 2003. The use of restraint/seclusion was very rare (1.71/10,000 per year). The use further decreased after a law reform set even stricter criteria for coercive measures (‘acceptable only to stop violent behaviour or prevent imminent violence’) (25). Stewart et al. (26) evaluated data collected via a database collecting data on intrusive measures used in a child and youth mental health treatment centre serving a 17-county catchment area in Ontario, Canada. They reported the use of chemical restraint (48.8% of patients) physical restraint (42.3%) or secure isolation (39.3%) during treatment. Developmental disabilities increased the risk for coercive measures (26). Green-Hennessy et al. (27) reported on the use of seclusion/restraint in all US residential treatment centres for children and adolescents via a federally-sponsored survey of mental health services. With a high response rate (88.8%), they found 82% of institutions to use seclusion/restraint. However, no data on the percentage of affected residents was available (27).

To sum up, the use of coercive measures appears to vary greatly on an international level. However, it is difficult to draw international comparisons between rates of coercive measures, since the definition of coercive measures and the regulatory framework varies widely.

REDUGIA assessed the impact of the 2017 law reform in Germany on the prevalence of challenging behaviour, coercive measures and employee stress. This article reports findings from the comparison between 2017 (baseline, pre-reform) and 2019 (follow-up, post-reform) data.

We expected to see a positive relationship of challenging behaviour with coercive measures and with employee stress. We furthermore expected to find a decrease in coercive measures from 2017 to 2019. We formed this hypothesis for two reasons: Firstly, the public focus on the topic due to the amendment of §1631 b might prompt staff and supervisors to re-think the necessity of those types of measures—especially ones that weren't previously classed as coercive measures. And secondly, the paperwork and time required for going through the courts might also prove an obstacle that may have fueled efforts to prevent coercive measures.

And we finally expected an increase in challenging behaviour due to the aforementioned expectation of lower rates of coercive measures in response to challenging behaviour.

## Methods

For the REDUGIA survey, we devised a 48-item questionnaire on structural characteristics of the residential institutions as well as on characteristics of the residents, challenging behaviour, coercive measures and employee stress. All data were self-reported by either management or staff at the participating institutions. We collected pseudonymized information ensuring that the individual institutions or individual residents are not identifiable.

### Challenging Behaviour (cB)

Institutions reported data on the number of residents displaying different forms of challenging behaviour as well as the frequency of so-called critical behaviours (aggression towards other residents, self-injurious behaviour, injury of staff members, destructive behaviour) in the last 14 days (for details, see Table 2). The *Index cB* describes the proportion of residents with challenging behaviour by dividing the number of residents with cB by the total number of residents.

### Coercive Measures (cM)

Institutions reported data on the frequency of different kinds of coercive measures as well as the number of children subjected to each type of coercive measure (for details, see Table 3). The *Index cM* represents the proportion of residents subject to coercive measures by dividing the number of residents with cM by the total number of residents.

### Employee Stress

We assessed different indicators of employee stress: physical assaults and uses of protective clothing in the last 14 days and instances of sick leave and requests for transferral or quitting the job due to challenging behaviour in the last 12 months.

Some providers operating more than one residential institution reported data for all of their institutions in one questionnaire. We received *N* = 43 questionnaires for the follow-up assessment and only included those with a corresponding baseline questionnaire in the analysis.

The study was approved by the ethics committee of the medical faculty of the University of Würzburg (study number 227/17).

### Statistical Analysis

We performed all analyses with IBM SPSS Statistics Version 26. Descriptively, we report frequencies, percentages, sum scores and means with standard deviations. For the analysis of the relationships between challenging behaviour with coercive measures, challenging behaviour with employee stress and coercive measures with employee stress, we calculated regression models or Pearson correlations. Changes between baseline (2017) and follow-up (2019) measurements were assessed with *t*-tests for dependent measures. The significance level was set at *p* = 0.05. We adjusted for multiple testing as follows: for the main pre-post comparisons (*Index cM, Index cB* and the 4 indicators of ES), the significance level was set to 0.008. For the correlations (*Index cM* with *Index cB*; *Index cM* and *Index cB* with the 4 indicators of ES), the significance level was set to 0.005. All other exploratory comparisons are uncorrected.

## Results

### Results From Follow-Up Assessment and Comparison Pre and Post Reform

43 of the 51 institutions (i.e., questionnaires) participating in the baseline evaluation in 2017 also provided data for the follow-up assessment in 2019 (84%). Descriptively, the overall total number of children, adolescents and young adults with IDD living in institutions remained stable. 13 institutions had at least one intensive group. The number of regular groups increased, whereas the number of intensive groups decreased. Conversely, the number of residents in intensive groups and under restrictive placements decreased despite a higher number of residents for which permissions for use of coercive measures according to §1631b BGB were obtained from the family court. Overall, groups became smaller while intensive groups had a slightly higher number of inhabitants than in 2017. The mean number of intensive and regular groups per institution remained stable (all *p*s > 0.923). The mean number of residents in total and per regular or intensive group as well as those placed under §1631b also was constant (all *p*s > 0.112). There were no differences in the number of full-time position equivalents (*p* = 0.119; Table 1).

**Table 1:** Characteristics of occupancy in 2017 und 2019 summarised over all *n* = 43 institutions participating in the follow-up assessment.

	**2017**				**2019**				
	**Total number**	**m per institution (sd)**	**Min**	**Max**	**Total number**	**m per institution (sd)**	**Min**	**Max**	***p***
Number of groups	255.5	5.9 (5.5)	1	29	215^*^	5.1 (3.8)	1	15	0.115
Among those: intensive groups (if any)	34	2.13 (1.0)	1	4	25	1.92 (1.1)	1	4	1.000
Among those: regular groups	221.5	5.8 (5.4)	1	27	190^*^	4.9 (3.7)	1	14	0.923
Number of residents	1,661	38.6 (32.0)	6	131	1,673	38.9 (34.5)	5	144	0.920
Among those: in intensive groups	172.5	11.5 (5.0)	6	24	131	10.1 (5.7)	4	20	0.728
Among those: in regular groups	1488.5	38.2 (32.1)	6	131	1,542	38.6 (33.8)	5	134	0.807
Among those: restricted placements	102	2.4 (6.3)	0	26	50	1.2 (3.6)	0	18	0.128
Permission obtained from family court (§1631b BGB)	146	3.4 (6.7)	0	26	229	5.5 (11.0)	0	56	0.112
Group size									
Size of intensive groups		5.6 (1.6)	1.8	7.0		5.6 (2.3)	2.5	12	1.000
Size of regular groups		7.2 (2.3)	2.2	13.3		7.5 (2.7)	0.87	21	0.416
Full-time position equivalents	1663.2	38.7 (40.6)	6	249.6	1756.4	42.8 (43.0)	7	247	0.119

* *One institution reported 49 groups (including 3 intensive groups) for N = 52 residents. This obvious error could not be resolved. We therefore excluded this institution for the calculation of the mean number of groups and counted only the 3 intensive groups of the institution towards the total number of groups overall*.

### Challenging Behaviour

Institutions did not report any changes in the *Index cB* (*p* = 0.446) or the proportion of children per institution displaying various forms of challenging behaviour (all *p*s > 0.220, see Table 2).

**Table 2:** Mean proportion of residents displaying challenging behaviour in the last 14 days.

**Type of challenging behaviour**	**Proportion of children and adolescents (%)**		
	**2017**	**2019**	***p***
Any severe challenging behaviour	14.1	9.6	0.771
Stereotyped behaviour	20.3	19.2	0.736
Destructive behaviour	16.9	14.1	0.214
Self-injurious behaviour	15.5	12.6	0.220
Aggression	13.3	15.0	0.390
Motor restlessness	12.7	13.3	0.805
Risk of running away	11.4	9.8	0.449
Excessive screaming	8.8	9.6	0.615
Changes in circadian rhythm	3.7	4.5	0.576
Other	2.2	2.6	0.780

**Figure 1:**
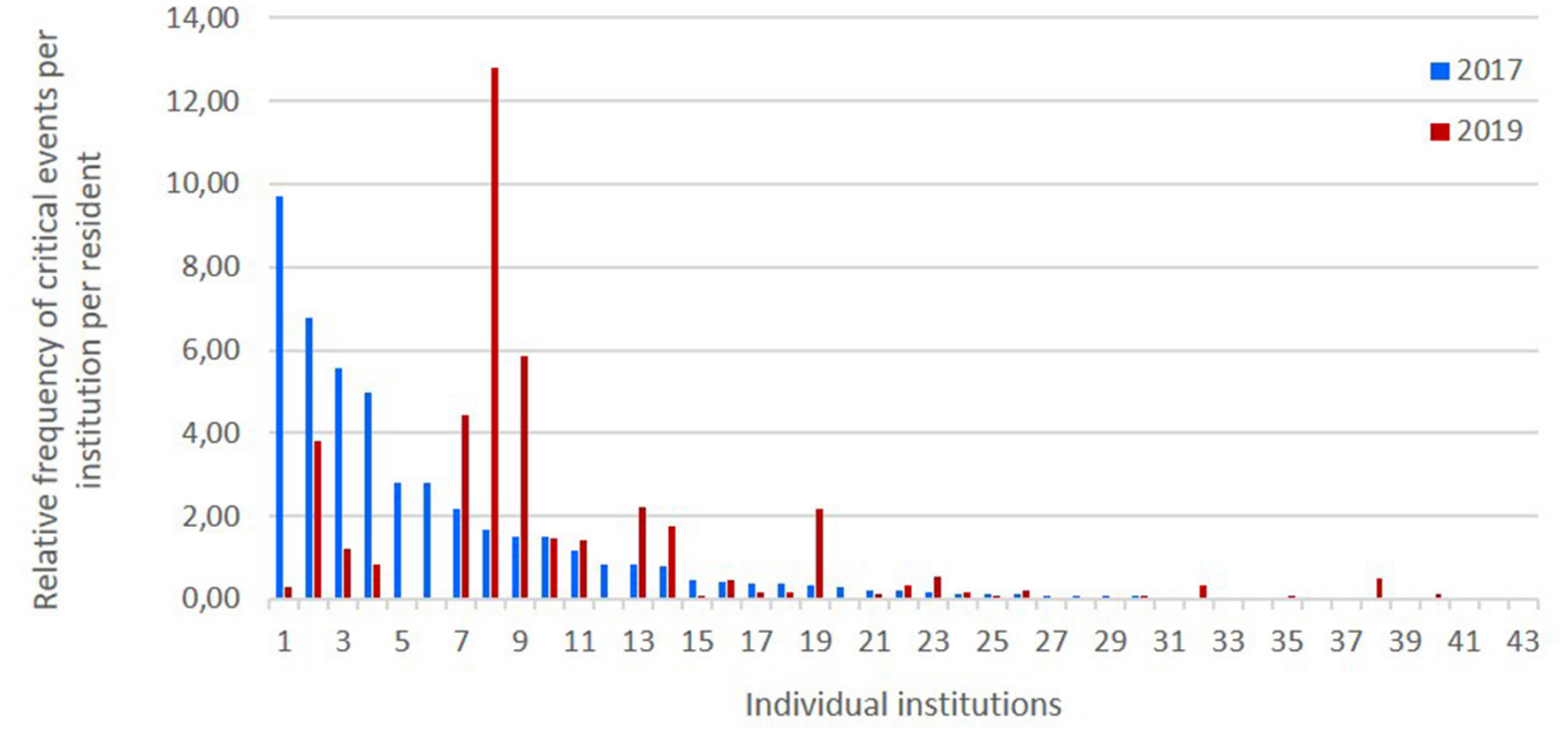
Relative frequency of critical events per institution per resident within 14 days in 2017 und 2019. The x-axis displays the institutions in descending order according to the sum of critical events per resident in 2017 (baseline assessment).

Figure 1 displays the relative frequency of critical events per institution per resident within 14 days in 2017 und 2019. Within institutions, there are fluctuations in the number critical events in that time period.

### Coercive Measures

**Figure 2:**
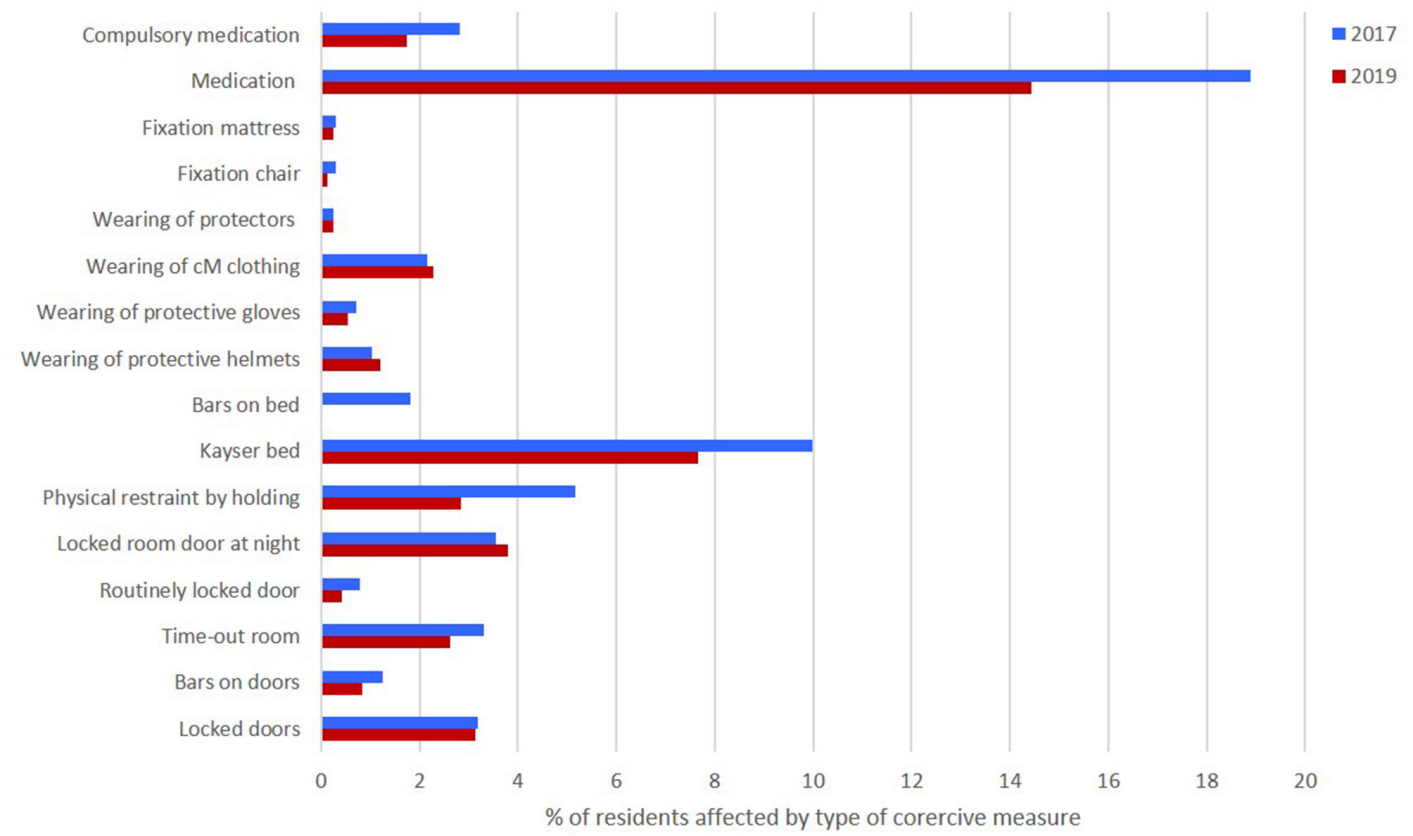
Overall percentage of children affected by any type of coercive measure.

Overall, only a comparatively small proportion of children living in residential institutions were subject to coercive measures (Figure 2) according to the institutions' self-report. Considering that children with severe challenging behaviour experienced multiple kinds of coercive measures, the true percentage is likely lower.

There was no change in the *Index cM* between baseline and follow-up (*p* = 0.241). Only compulsory medication decreased between measurements (*p* = 0.028). There were trend-level decreases in the number of children sleeping in a Kayser bed (*p* = 0.051) and restraint by holding (*p* = 0.070). All other coercive measures remained at the 2017 level (all *p*s > 0.111) (see Table 3).

**Table 3:** Mean proportion of children and adolescents per residential institution affected by coercive measures in the last 14 days.

**Type of coercive measure**	**Proportion of children and adolescents (%)**		
	**2017**	**2019**	***p***
Kayser bed	14.4	10.5	0.051
Physical restraint by holding	7.9	5.4	0.070
Time-out room	3.6	2.8	0.578
Locked room door at night	3.5	3.2	0.734
Locked doors	3.2	3.1	0.904
Wearing of restrictive clothing	2.3	3.6	0.453
Bars on doors	1.4	0.8	0.282
Bars on bed	1.3	0.0	0.111
Compulsory medication^*^	3.4	1.9	0.028
Wearing of helmets^**^	1.1	1.5	0.647
Routinely locked door	0.8	0.5	0.169
Wearing of gloves^**^	0.8	0.6	0.369
Fixation mattress	0.5	0.4	0.530
Fixation chair^**^	0.4	0.1	0.166
Wearing of protectors^**^	0.2	0.3	0.991

* *only emergency use medication*.

** *Coercive measures that were not regulated by §1631b prior to the amendment*.

**Figure 3:**
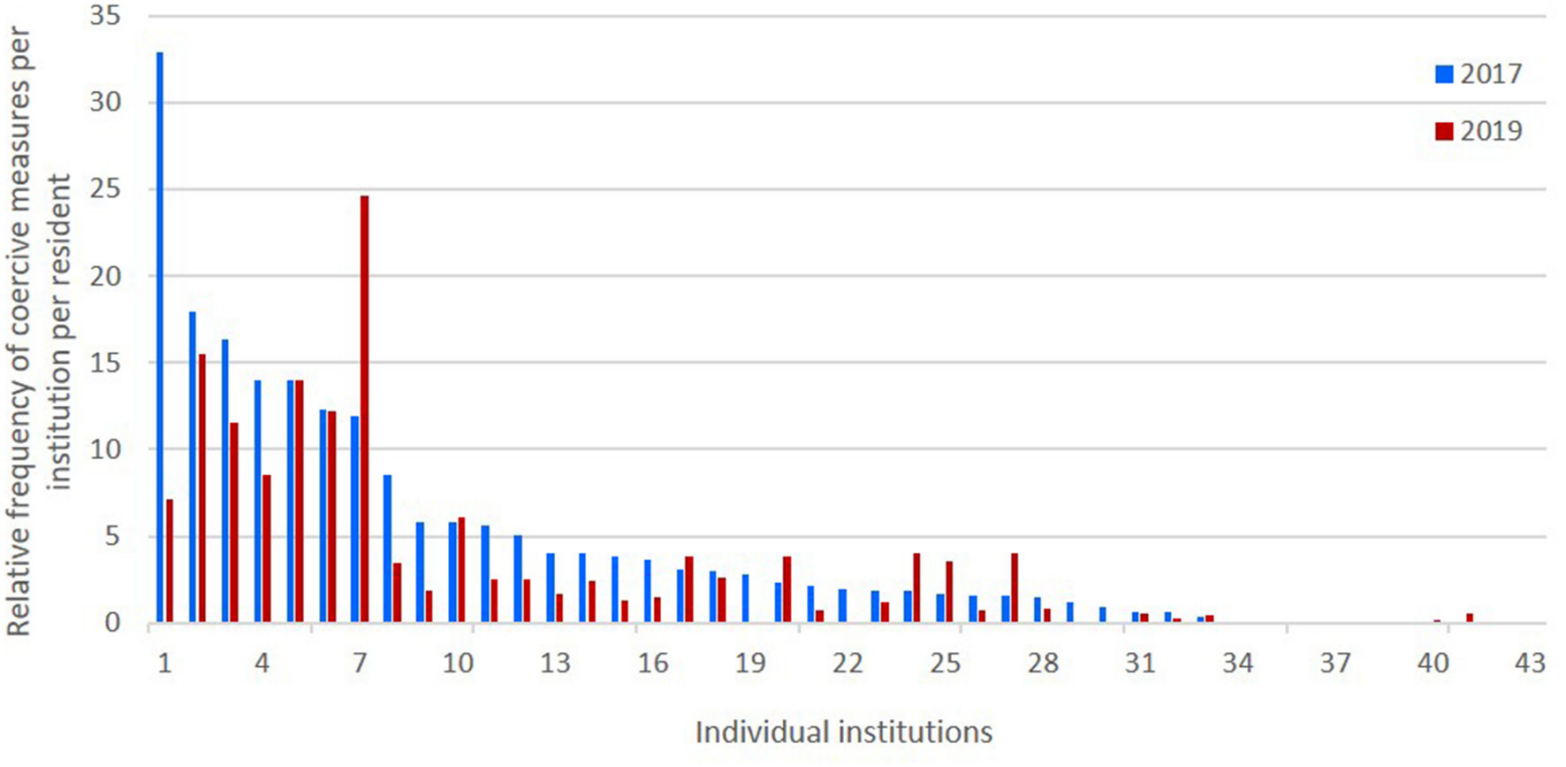
Relative frequency of coercive measures per institution per resident within 14 days in 2017 und 2019. The x-axis displays the institutions in descending order according to the sum of cM per resident in 2017 (baseline assessment).

Figure 3 shows the relative frequency of coercive measure per institution per resident within 14 days in 2017 und 2019.

### Employee Stress

Institutions did not report changes in any indicators of employee stress from baseline to follow- up, all *p*s > 0.323. Descriptively, we observed a slight increase in the absolute number of full-time position equivalents and decreases in the total number of physical assaults, the need for protective clothing for employees and the frequency of employees requesting a transfer or quitting the job due to challenging behaviour (Table 4).

**Table 4:** Staff characteristics and employee stress (absolute number of incidents and mean relative frequency per full-time position equivalent) in *n* = 43 institutions participating in the follow-up assessment.

	**2017**			**2019**			
	**Absolute**	**Relative (%)**	**Institutions without** **N (%)**	**Absolute**	**Relative**	**Institutions without** **N (%)**	***p***
Full-time position equivalents	1663.2			1756.4			
Physical assault (last 14 days)	617	30.8	17 (39.5)	409	26.5%	16 (37.2)	0.702
Protective clothing for employees (last 14 days)	107	3.2	41 (95.3)	39	0.9%	41 (95.3)	0.323
Sick leave due to cB (last 12 months)	78,5	6.3	21 (48.8)	84	5.2%	21 (51.2)	0.630
Number of employees requesting a transfer or quitting the job due to cB (last 12 months)	31	2.4	27 (62.8)	19	1.5%	29 (67.4)	0.360

### Relationship Between cB, cM and ES

**Figure 4:**
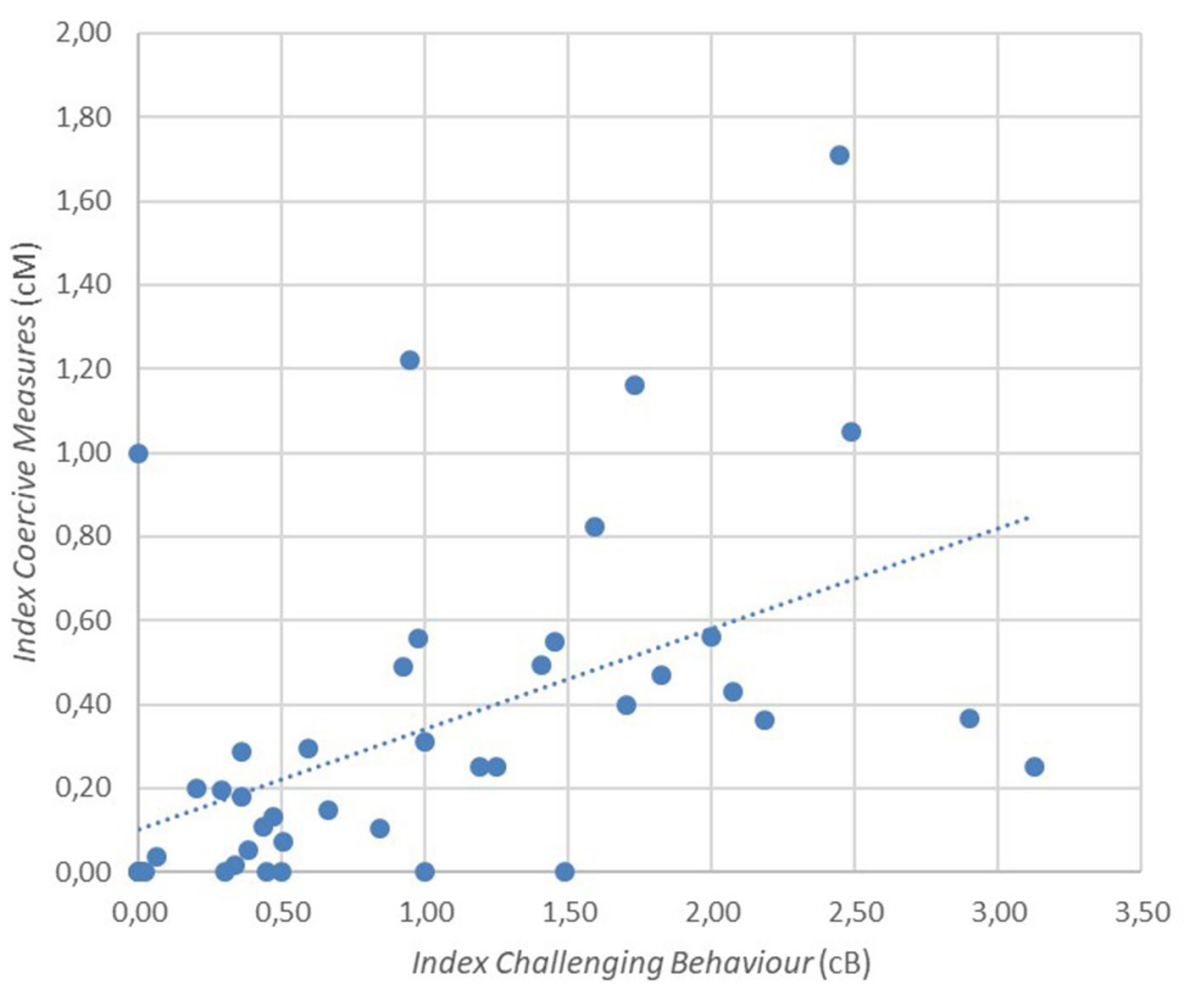
Relationship between the Index cB and the Index cM, *r* = 0.519, *p* < 0.001.

At follow-up, the *Index cB* correlated significantly with the *Index cM* (*r* = 0.519 *p* < 0.001, Figure 4). Regarding links to employee stress, the *Index cB* correlated positively with the frequency of sick leave on a trend level (*r* = 0.322, *p* = 0.037) and with physical attacks on employees (*r* = 0.552, *p* < 0.001). The *Index cM* also correlated positively with the frequency of sick leave on a trend level (*r* = 0.340, *p* = 0.028) and with physical attacks on employees (*r* = 0.492, *p* = 0.001).

## Discussion

Our study was the first to systematically examine challenging behaviour, coercive measures and employee stress in residential institutions for children, adolescents and young adults with IDD in a German federal state (Bavaria). We furthermore assessed changes in the rates of challenging behaviour, coercive measures and employee stress after the law reform addressing the definition and use of coercive measures in children and adolescents. All data were self-reported by the participating institutions.

In summary, REDUGIA delivered a detailed picture on the topic of coercive measures in institutions for children, adolescents and young adults with IDD. While the findings do not support the worrying accusations of broadly and inadequately applied coercive measures, the data represent a benchmark for future endeavours to further reduce coercive measures. Overall, only a comparatively small proportion of children in residential institutions in Bavaria was affected by coercive measures. With the exception of Kayser beds (10% in 2017, 7.7% in 2019), no coercive measure was employed for more than 5% of the children. Medication for psychiatric disorders (18.9% in 2017, 14.4% in 2019) is not strictly speaking a coercive measure, since it is medically necessary and does not restrict freedom of movement. Considering that children with severe challenging behaviour experienced multiple types of coercive measures, the true percentage of children who are affected by coercive measures at all is likely lower.

Both pre and post reform, coercive measures correlated positively with challenging behaviour, confirming our hypothesis. This points to the primary use of coercive measures as an interventional response towards challenging behaviour, which fits with the existing literature. Nonetheless, challenging behaviour is amenable to interventions other than coercive measures (28). Standardised analyses of behaviour have shown high success rates in identifying and addressing the causes of challenging behaviour (29) and the Triple P Stepping Stones program for parents of children with disabilities is highly effective in reducing challenging behaviour and caregiver stress (30, 31). International guidelines for challenging behaviour in intellectual disability recommend a thorough analysis of living conditions and the function of behaviour for addressing self-injurious behaviour (1, 32).

We could also confirm our hypothesis of a positive correlation between challenging behaviour and employee stress. This finding corresponds with the existing literature reporting a link of challenging behaviour to caregiver stress and quality of life (8). Subjectively, research indicated that staff in institutions for people with IDD rated challenging behaviour as one of the major stressors (9, 10). However, using objective measures of exposure to challenging behaviour, the picture is less clear. Flynn et al. (33) found no association of work-related well-being with the exposure to aggressive challenging behaviour. The authors discussed the influence of different measures of exposure to challenging behaviour and hypothesise that ‘emotional exhaustion and positive work motivation are more substantially influenced by working environment than the other variables’ (33). In addition to differences in the definition and assessment of challenging behaviour, the definition of ES is also relevant when looking at the link between challenging behaviour and ES. In our study, we focused on physical (physical assault, need for protective clothing) as well as indirect indicators of stress (instances of sick leave, requests for transferral or giving notice due to challenging behaviour). We did not assess the psychological impact of challenging behaviour and coercive measures. Especially for psychological well-being, coping style and personality may be more relevant for the degree of experienced (di)stress. Future research should address this question. Institutions with higher rates of challenging behaviour had a higher staff to patient ratio probably indicative of the higher severity of challenging behaviour in the respective group of children and adolescents. Nevertheless, employee stress was rated higher in those institutions. This may also be due to 3 of the 4 indicators of employee stress—physical assaults, use of protective clothing and instances of sick leave—potentially being direct results of challenging behaviour in the form of aggression. However, it is also possible that the additional staff members did not work in the positions of greatest exposure.

Considering the effects of the law reform, we could not confirm our hypotheses of an increase in challenging behaviour or a decrease in coercive measures. The percentage of children, adolescents and young adults displaying challenging behaviour at follow-up was comparable to baseline. The number of children with severe challenging behaviour was comparatively low both in 2017 (14.1%) and 2019 (9.6%). After the law reform, applications to family courts for permission for the use of coercive measures increased by 57%. However, the percentage of children affected by coercive measures remained roughly the same. This may indicate that even before the requirement of permission from family courts, the institutions had likely carefully examined the necessity of coercive measures before initiating them. However, this does not imply that challenging behaviour necessitates coercive measures *per se*. Employees likely find themselves in situations, in which they have no alternative to using coercive measures to address challenging behaviour, due to e.g., structural conditions or a lack of resources. Creating judicial obstacles such as the law reform cannot address those underlying issues and therefore cannot by itself reduce coercive measures. To meaningfully reduce coercive measures, concomitant changes in pedagogic and therapeutic concepts and the resources at the disposal of the institutions are required. While it is possible that due to the sensitive nature of the topic institutions may have been incentivized to conceal the true frequency of coercive measures, the assured anonymity of the published data and the heterogeneity of reported coercive measures rates between institutions lends credibility to the statements made by the respondents. Furthermore, we have visited 20 institutions for qualitative interviews (data not reported here) that supported the validity of the surveys.

We found a pronounced heterogeneity between institutions in term of the rates of challenging behaviour and coercive measures. Challenging behaviour and coercive measures concentrated in approximately one third of the participating institutions. Around 60% of the institutions reported hardly any or no challenging behaviour or coercive measures. This fits with the observation in our clinical routine that only highly specialised institutions accept residents who display challenging behaviour, especially aggressive behaviour. Furthermore, this pattern confirms that a large proportion of youth with IDD does not display any kind of challenging behaviour. In fact, only 14.1% (T1) and 9.6% (T2) reported any severe challenging behaviour. While this appears low compared to the estimated 52% reported in students attending specialised schools for IDD in Bavaria in the study by Dworschak and colleagues, these numbers fit with population-based estimates of the prevalence of challenging behaviour in people with IDD (34). In the Emerson study, ‘informants were instructed to complete these sections if the person showed that form of challenging to the extent that it was considered by them to constitute a serious management problem’. In contrast, Dworschak and colleagues used a composite score from a comprehensive questionnaire inquiring after 33 specific challenging behaviours, whereas REDUGIA asked about more generally about the *number of residents displaying severe challenging behaviour* in addition to inquiring about eight specific types of challenging behaviour. It is likely that caregivers did not consider some of the behaviours scored in the study by Dworschak et al. as severe challenging behaviour. It can however not be ruled out that the institutions without challenging behaviour and coercive measures have a different concept or differing staff qualifications that prevent the occurrence of challenging behaviour and subsequently the need for coercive measures. It would be of great interest to further investigate institutions with low rates of challenging behaviour and coercive measures. We did not collect data on residents' degree of impairment, comorbid diagnoses, staff qualifications and details on quality control measures. While it is possible that those institutions with low challenging behaviour and low coercive measures had less severely afflicted residents, it is also possible that there are e.g., structural or personnel differences that prevent challenging behaviour or allow for an approach to challenging behaviour without coercive measures. Especially considering the small numbers of children in most institutions, it can have a considerable impact on the frequency of critical events and thus freedom-restricting measures if even a few residents with severe challenging behaviour move into or out of an institution. Another factor in the management of challenging behaviour is the level of care for the severely affected residents. One such resident being approved for or stripped of 1:1 care from a designated member of staff can lead to significant changes. Furthermore, our survey only inquired after events of the last 14 days. So even temporary changes such as e.g., a hospital stay, can influence the numbers.

In addition to a general lack of expertise in the field of IDD and of therapeutic options, the dichotomization into ‘regular’ and ‘intensive’ institutions leads to an increasing deficit in the availability of suitable care for children, adolescents and young adults with IDD. Given the political and societal measures to encourage the inclusion of individuals with developmental disorders into all societal contexts, the demand for regular institutions is declining compared to more intensive institutions. At the same time, medical advancements ensure a substantially higher survival rate of children with complex developmental disorders and severe somatic impairments that require specialised care. Hence, ‘intensive’ institutions have by far more applications than they can actually accept and thus children with the most severe behavioural issues cannot be placed. In fact, those children with the highest needs generally have the longest waiting periods until placement or cannot be placed at all. Often, those children remain in psychiatric clinics and are—upon reaching adulthood—transferred to the adult departments, where in many cases they are hospitalised for many years. Before the reform of §1631b BGB, there were only 102 official restricted placements in Bavaria. The surveyed institutions had obtained permissions from a court for the use of coercive measures for 151 children, adolescents and young adults, indicating that the need for coercive measures potentially exceeds the number of official restricted placements. However, even if permission was obtained, this does not automatically mean that coercive measures were used. Outside of a restricted placement, the use of coercive measures prior to the reform was exclusively regulated via custodial consent. It has frequently been discussed whether the limited number of residential placement options for children, adolescents and young adults with IDD—especially those displaying challenging behaviour—puts pressure on custodians to agree to the use of coercive measures in order to avoid losing the child's placement.

In addition, challenging behaviour and coercive measures lead to the stigmatisation of both the children and their caregivers. Children with challenging behaviour are viewed as hard to adequately take care of. Aggressive behaviour presents a danger to caregivers, who may feel helpless in the face of those behavioural issues—especially if they lack alternatives to coercive measures to address the issues. At the same time, the use of coercive measures is often judged as unjustified and ‘the easy way out’ by external observers, and this creates additional pressures for caregivers. The higher rate of coercive measures in ‘intensive’ institutions as a consequence of caring for more severely affected children, adolescents and young adults with higher levels of aggression has repeatedly led to accusations of malpractice against those institutions. Especially children, adolescents and young adults with aggressive behaviour issues require specific conditions to thrive. Institutions require the resources—both structural as well as in terms of staff training and therapeutic-pedagogic concepts—to provide viable alternatives to coercive measures to address and ideally prevent challenging behaviour. While law reforms restricting access to coercive measures are an important first step, only the concurrent development of innovative concepts will lead to reductions in challenging behaviour and coercive measures in the long term. REDUGIA provides extensive data on the status quo. Future research should focus on systematically evaluating preventive measures and interventions.

## Limitations

The reliance on institutions' self-report regarding the use of coercive measures is the most serious limitation of the REDUGIA project, since such a sensitive topic can lead to socially desirable answers. We tried to address this issue by assuring institutions of anonymity after the follow-up data collection. Still, we cannot rule out the possibility that instances of coercive measures were omitted.

Some providers running multiple residential institutions reported on all of their institutions in one questionnaire. This may have introduced a bias in the data if institutions were very dissimilar.

There are several limitations within the composition of the questionnaire itself. Future surveys should include items assessing a) the number of residents displaying no challenging behaviour, b) the number of residents not affected by coercive measures, c) the frequency of each type of challenging behaviour, d) co-occurring coercive measures after one incident e) the number of persons on staff in addition to the full-time position equivalents, f) psychological employee stress and g) coping strategies and successful strategies for dealing with challenging behaviour without coercive measures.

Lastly, our results pertain to conditions in residential institutions in the state of Bavaria. It is unclear to what extent they can be generalised across the whole of Germany. More research is needed to provide a comprehensive picture.

## Conclusion

There is a distinct link between coercive measures and challenging behaviour. Addressing the underlying causes of challenging behaviour is therefore key to reducing the need for coercive measures.

Challenging behaviour and the use of coercive measures only occurred in one third of participating institutions. There is reason to believe that this is due to only a minority of institutions admitting residents with known aggressive behaviour. A broader dissemination of specialised knowledge especially regarding aggressive behaviour in people with IDD may create more residential placement options for these complex cases.

The amendment of §1631b BGB mandating permission from family courts for the use of coercive measures did not lead to a decrease in the use of coercive measures. This points to a responsible use of coercive measures by residential institutions for children, adolescents and young adults with IDD.

## Data Availability Statement

The raw data supporting the conclusions of this article will be made available by the authors, without undue reservation.

## Ethics Statement

The studies involving human participants were reviewed and approved by Ethics Committee of the medical faculty of the University of Würzburg (study number 227/17). Written informed consent for participation was not required for this study in accordance with the national legislation and the institutional requirements.

## Author Contributions

EW, CR, and MR contributed to conception and design of the study. JG and EW performed the statistical analysis. JG wrote the first draft of the manuscript. All authors contributed to manuscript revision, read, and approved the submitted version.

## Funding

This study was funded by the Bavarian State Ministry of Family, Employment and Social Affairs (StMAS). This publication is supported by the Open Access Publication Fund of the University of Wuerzburg.

## Conflict of Interest

The authors declare that the research was conducted in the absence of any commercial or financial relationships that could be construed as a potential conflict of interest.

## Publisher's Note

All claims expressed in this article are solely those of the authors and do not necessarily represent those of their affiliated organizations, or those of the publisher, the editors and the reviewers. Any product that may be evaluated in this article, or claim that may be made by its manufacturer, is not guaranteed or endorsed by the publisher.
